# Genetic variants of genes involved in thiopurine metabolism pathway are associated with 6-mercaptopurine toxicity in pediatric acute lymphoblastic leukemia patients from Ethiopia

**DOI:** 10.3389/fphar.2023.1159307

**Published:** 2023-05-09

**Authors:** Awol Mekonnen Ali, Haileyesus Adam, Daniel Hailu, Ephrem Engidawork, Rawleigh Howe, Teferra Abula, Marieke J. H. Coenen

**Affiliations:** ^1^ Department of Pharmacology and Clinical Pharmacy, School of Pharmacy, College of Health Sciences, Addis Ababa University, Addis Ababa, Ethiopia; ^2^ Department of Pediatrics and Child Health, School of Medicine, College of Health Sciences, Addis Ababa University, Addis Ababa, Ethiopia; ^3^ Armauer Hansen Research Institute, Addis Ababa, Ethiopia; ^4^ Department of Human Genetics, Radboud Institute for Health Sciences, Radboud University Medical Center, Nijmegen, Netherlands

**Keywords:** acute lymphoblastic leukemia, xanthine dehydrogenase, neutropenia, pharmacogenetics, 6-mercactopurine

## Abstract

**Introduction:** Genetic variation in the *thiopurine S-methyltransferase* (*TPMT*) gene by and large predicts variability in 6-mercaptopurine (6-MP) related toxicities. However, some individuals without genetic variants in *TPMT* still develop toxicity that necessitates 6-MP dose reduction or interruption. Genetic variants of other genes in the thiopurine pathway have been linked to 6-MP related toxicities previously.

**Objective:** The aim of this study was to evaluate the effect of genetic variants in *ITPA*, *TPMT*, *NUDT15*, *XDH*, and *ABCB1* on 6-MP related toxicities in patients with acute lymphoblastic leukemia (ALL) from Ethiopia.

**Methods:** Genotyping of *ITPA*, and *XDH* was performed using KASP genotyping assay, while that of *TPMT*, *NUDT15,* and *ABCB1* with TaqMan^®^ SNP genotyping assays. Clinical profile of the patients was collected for the first 6 months of the maintenance phase treatment. The primary outcome was the incidence of grade 4 neutropenia. Bivariable followed by multivariable cox regression analysis was performed to identify genetic variants associated with the development of grade 4 neutropenia within the first 6 months of maintenance treatment.

**Results:** In this study, genetic variants in *XDH* and *ITPA* were associated with 6-MP related grade 4 neutropenia and neutropenic fever, respectively. Multivariable analysis revealed that patients who are homozygous (CC) for *XDH* rs2281547 were 2.956 times (AHR 2.956, 95% CI = 1.494–5.849, *p* = 0.002) more likely to develop grade 4 neutropenia than those with the TT genotype.

**Conclusion:** In conclusion, in this cohort, *XDH* rs2281547 was identified as a genetic risk factor for grade 4 hematologic toxicities in ALL patients treated with 6-MP. Genetic polymorphisms in enzymes other than *TPMT* involved in the 6-mercaptopurine pathway should be considered during its use to avoid hematological toxicity.

## 1 Introduction

Globally, acute lymphoblastic leukemia (ALL) is the most common childhood cancer (Johnston et al., 2021). In Ethiopia, the annual incidence of childhood cancers has been estimated between 3,707 and 6,000 cases, with leukemia being the most prevalent cancer (29%) (Aziza et al., 2013; Memirie et al., 2018). Over 90% of patients with childhood ALL in developed countries can be cured as a result of risk-adapted and good systemic chemotherapy. However, treatment outcomes in resource-limited countries are lower, with cure rates ranging from 40% to 70% depending on where the patient is treated (Bahnassy et al., 2020; Tandon, 2020).

In addition to its role in other treatment phases, 6-mercaptopurine (6-MP) is the backbone of the maintenance phase of ALL treatment (Lee et al., 2017). Dose intensity of 6-MP is a key component determining treatment outcome of ALL patients (Relling et al., 1999). Despite good treatment outcomes the downside of 6-MP treatment is that it can induce severe life-threatening hematological and hepatotoxicity. Myelosuppression is particularly complicated when it is accompanied by a severe infection which leads to dose reductions, drug discontinuation, or even treatment interruption and death (Mao et al., 2021). To avoid myelosuppression, 6-MP is titrated to the desired absolute neutrophil count (ANC) value and therefore dose adjustments are routinely made (Karol et al., 2021).

Pharmacogenomics can play a role in increasing the efficacy and reducing toxicity of 6-MP in ALL patients by assisting optimal dose selection for the individual patient. 6-MP is a pro-drug, which requires extensive metabolism to the active metabolite 6-thioguanine. Genetic variants in drug metabolizing enzymes and transporters are known to alter 6-MP treatment outcomes (Jantararoungtong et al., 2021). For instance, it is well established that genetic variants in the thiopurine S-methyltransferase (TPMT) gene are associated with 6-MP treatment intolerance (Wang and Weinshilboum, 2006). To avoid toxicities, 6-MP dose adjustment can be made based on genetic variation in *TPMT*. TPMT*2, TPMT*3B, and TPMT*3C variants alter the tertiary structure of the *TPMT* protein, leading to instability and reduced catalytic activity (Chen et al., 2021). Patients that show an intermediate TPMT enzyme activity are advised to start with a reduced dose (30%–50%) and a 10 fold reduction or alternative treatment is recommended for patients with a complete TPMT deficiency (Bertholee et al., 2017). More recently, *NUDT15* rs116855232 and rs746071566 variants have been linked to 6-MP intolerance (Chiengthong et al., 2016; Walker et al., 2019) and treatment guidelines have also been developed for this gene (Relling et al., 2019). *NUDT15* (rs116855232) mutation adversely affects the protein stability of the enzyme, leading to rapid degradation (Valerie et al., 2016). Whereas, rs746071566 variant reduces NUDT15 activity (Walker et al., 2019). Besides *TPMT* and *NUDT15,* other genes in the 6-MP metabolism have also been investigated in relation to development of side effects. For example, genetic variants in *ITPA* (rs1127354 and rs7270101) reduce enzymatic activity which may increase toxicity risk due to an accumulation of potentially toxic metabolite thioITP (Marinaki et al., 2004; Maeda et al., 2005; Atanasova et al., 2007). A more recent study showed that genetic variants in *XDH* were associated with thiopurine metabolism and thiopurine related toxicities, including neutropenia, hepatotoxicity, and treatment interruption (Choi et al., 2019). The ATP-binding cassette sub-family B member 1 (*ABCB1*) gene encodes for P-glycoprotein. ABCB1 overexpression could be accountable for therapy failure or relapse while the reduced activity can lead to more severe 6-MP toxicity (Milosevic et al., 2018).

In the Caucasian population, genetic variants for *TPMT* have been identified in about 11% (Zeglam et al., 2015) of the population. On the other hand, variants in *NUDT15* are rare with frequencies ranging from 0.2% (Coenen, 2019) to 2.4% (Schaeffeler et al., 2019). There is, however, a scarcity of pharmacogenetic studies focusing on other genes in the 6-MP metabolic pathway, especially in non-Caucasian populations. This study therefore aimed to investigate the frequency of variants coding for drug metabolizing enzymes (*TPMT*, *ITPA*, *NUDT15*, and *XDH*), and transporter (*ABCB1*) in the thiopurine metabolic pathways in Ethiopian children with ALL and their association with 6-MP-related adverse events.

## 2 Materials and methods

### 2.1 Patient recruitment and 6-MP treatment

This study included 160 pediatric patients under the age of 12 years at the time of diagnosis, who were treated from 2019 to 2021 at the pediatric oncology wards of Tikur Anbessa specialized hospital (TASH), which provides organized cancer care services. Patients were treated using a protocol for low- and middle-income countries (Hunger et al., 2009). The maintenance phase of this protocol includes daily oral 6-MP and weekly oral methotrexate (MTX). The patients also received monthly vincristine 1.5 mg/m^2^, 5 days of dexamethasone per month (6 mg/m^2^/d for 5 days/mo) and intrathecal MTX (age-adjusted dosing) until 2.5 years from the date of diagnosis. Trimethoprim/sulfamethoxazole (TMP) at a dose of 5 mg/kg/d 3 times per week was given for *Pneumocystis jirovecii* prophylaxis as co-medication. Patients were stratified into three groups based on a physical examination, age, initial white blood cell count (WBC), central nervous system (CNS) status, and early prednisolone response. Informed consent was obtained from all participants parents or guardians. The study was approved by the Institutional Review Board (IRB) of the College of Health Sciences, Addis Ababa University (021/18), Armauer Hansen Research Institute Ethical Review Committee (P051/18), and National Research Ethics Review Committee of the Federal Democratic Republic of Ethiopia.

### 2.2 Sample and data collection

EDTA whole blood samples were collected from ALL patients. For all patients, the following information was collected from medical records; demographic data, clinical presentation during diagnosis, complete blood count (CBC) at diagnosis, peripheral morphology, peripheral and bone marrow blast, liver, and kidney function test, and risk group. Clinical data such as CBC, fever, emergency admission, dose reduction, and drug discontinuation were collected for the first 6 months of the maintenance phase treatment. CBC was performed at a 4-week interval unless it was indicated for any clinical reasons.

### 2.3 DNA isolation and genotyping

Genomic DNA was extracted from 1 mL of whole blood using QIAamp Blood Midi Kit (Qiagen GmbH, Hilden, Germany). DNA quality was checked using gel electrophoresis and NanoDrop™ ND-2000c Spectrophotometer.

Genotyping of SNP rs1127354 and rs7270101 in *ITPA* and rs2281547 in *XDH* was performed using a KASPar-On-Demand (KOD) assay (LGC Genomics, Hoddesdon, United Kingdom) as described previously (Vos et al., 2016), with little modification. The final volume for each reaction was 5 µL containing 1 µL of DNA, 2.5 µL of KASP 5000 V4.0 Low ROX, 0.0625 µL of the KASPar assay (40x), and 1.44 µL of MilliQ grade water. The PCR conditions consisted of an initial denaturation at 94°C for 15 min, followed by 10 cycles at 94°C for 20 s and annealing/extension at 61°C for 60 s including a drop of 0.6°C for each cycle. This was followed by 26 cycles of denaturation at 94°C for 10 s and annealing/extension at 55°C for 60 s, followed by 12 cycles of denaturation at 94°C for 20 s and annealing/extension at 57°C for 60 s. TPMT*2, TPMT*3B, TPMT*3C, *NUDT15* rs116855232, *NUDT15* rs746071566, and *ABCB1* rs1045642 variants were genotyped by TaqMan^®^ SNP Genotyping Assays (Assay ID number C__12091552_30 for TPMT*2, C__30634116_20 for TPMT*3B, C_____19567_20 for TPMT*3C, C_154823200_10 for rs116855232, and C___7586657_20 for rs1045642) as described elsewhere (Kumagai et al., 2019). The final reaction volume of 5 µL was prepared by mixing 1 µL of DNA, 2.5 µL of TaqMan^®^ Universal PCR Master Mix (2x; Applied Biosystems by Thermo Fisher scientific, Warrington, United Kingdom), 0.0625 µL of TaqMan^®^ SNP Genotyping Assay (40x; Applied Biosystems by Thermo Fisher Scientific), and 1.44 µL of MilliQ grade water. The PCR conditions included an initial stage at 95°C for 12 min and followed by a stage for 50 cycles step 1 at 92°C for 15 s and step 2 at 60°C for 90 s. For *NUDT15* rs746071566 custom designed assay (Id: ANDKGXA) was used. The final reaction volume of 10 µL was prepared by mixing 1 µL of DNA, 5 µL of TaqMan^®^ Universal PCR Master Mix, 0.25 µL of TaqMan^®^ SNP Genotyping Assay, and 3,75 µL of MilliQ grade water. The PCR conditions included an initial stage at 95°C for 10 min and followed by a stage for 50 cycles; step 1 at 95°C for 15 s and step 2 with 60°C for 60 s and post-read stage at 60°C.

### 2.4 Study outcomes

Myelotoxicity was graded according to Common Terminology Criteria for Adverse Events (CTCAE), version 4.0 (CTCAE, 2010). Accordingly, toxicity was classified as grade 4 when WBC <1,000/mm^3^; ANC <500/mm^3^; anemia <6.5 g/dL and thrombocytopenia <25,000/mm^3^. The primary outcome measure was the occurrence of grade 4 neutropenia within the first 6 months of the initiation of maintenance treatment. The secondary outcomes were, drug discontinuation, neutropenic fever, and early-onset grade 4 leukopenia/neutropenia. Early-onset leukopenia/neutropenia was defined as the occurrence of leukopenia/neutropenia during the first 60 days of the maintenance therapy (Zhou et al., 2018). Neutronic fever was defined as a grade 4 neutropenia with temperature of 38°C or greater.

### 2.5 Statistical analysis

Data were entered and analyzed using SPSS statistical package for Windows SPSS version 26. The Chi-square test was used to assess Hardy–Weinberg equilibrium (HWE). Descriptive statistics were used to examine the demographic characteristics, clinical profiles, and genotype frequencies of participants. Risk factor analysis and hazard ratios for grade 4 neutropenia, early-onset grade 4 leukopenia/neutropenia, treatment interruption, and neutropenic fever were calculated using cox proportional hazard regression analysis. Bivariable followed by multivariable analysis was computed with enter as the variable selection method. Results were expressed as hazard ratios (HRs) and 95% confidence intervals. For all test a *p*-value of 0.05 was considered significant. All genetic variants except *TPMT* and *NUDT15* were included in multivariable analysis to see the influence of the variants on hematotoxicity.

## 3 Results

### 3.1 Demographic, clinical characteristics and incidence of grade 4 hematological toxicity

In this study, 142 patients were included in the final analysis. Due to incomplete data, 18 study participants were not included in the final analysis. The clinical characteristics of the patients are shown in Table 1. The study group consisted of 92 (64.8%) males, and 50 (35.2%) females, and the mean age at diagnosis was 6.2 years old. Sixty-nine (48.6%) participants were categorized as standard risk, while the remaining 73 (51.4%) were high risk. The overall incidence of chemotherapy-induced grade 4 neutropenia was 52.8%. Fifty-six (39.4%) patients received full doses of chemotherapy as scheduled, while therapy was interrupted in 83 (58.5%) patients because of adverse events in the first 6 months of the maintenance treatment. The most frequent adverse event causing discontinuation was myelosuppression.

**TABLE 1:** Characteristics of the study population in the outpatient pediatric oncology department of TASH (*n* = 142).

		Number (%)
Child’s sex	Male	92 (64.8%)
	Female	50 (35.2%)
Child’s age (years)	≤ 6	79 (55.6%)
	> 6	63 (44.4%)
Risk group	Standard risk	69 (48.6%)
	High risk	73 (51.4%)
WBC at the beginning of maintenance therapy (cells/mm^3^)	< 4500	90 (63.4%)
	≥ 4500	52 (36.6%)
ANC at the beginning of maintenance therapy (cells/mm^3^)	< 2500	111 (78.2%)
	≥ 2500	31 (21.8%)
Neutropenia		75 (52.8%)
Neutropenic fever		44 (31%)
Treatment interruption		83 (58.5%)

Data are presented as number and percentage.

ANC, absolute neutrophil counts; 6-MP, 6-Mercaptopurine; WBC, white blood cell.

### 3.2 Genotyping and its association with the primary outcome

All genotype frequencies were according to HWE (*p* > 0.05). The overall allele frequencies of TPMT*3C, *ITPA* rs1127354, *ITPA* rs7270101, *XDH* rs2281547, and *ABCB1* rs1045642 variants were 0.35, 4.6, 11.6, 31, and 77.1%, respectively. No variant alleles were identified for TPMT*2, TPMT*3B, *NUDT15* rs116855232, and rs746071566. Table 2 shows a comparison of genotype and allele frequencies between patients who developed grade 4 neutropenia *versus* treatment tolerant patients. Patients carrying the *XDH* rs2281547 CC/TC genotype had a significantly higher incidence of grade 4 neutropenia (*p* = 0.01) as compared to TT genotype. Similar results were found for the allele analysis. The incidence of grade 4 neutropenia was higher in patients carrying *XDH* rs2281547 C allele (38.6%% vs. 22.3%, *p =* 0.003). The other allele frequencies did not differ significantly between patients with grade 4 neutropenia compared to treatment tolerant patients.

**TABLE 2:** Genotype and allele frequencies of candidate drug metabolizing enzymes and transporter genes by grade 4 neutropenia.

	SNP	Genotype/variant allele	Grade 4 neutropenia		*p*-value
			No, n (%)	Yes, n (%)	
Genotype frequency	TPMT*3C	TT	67 (100%)	74 (98.7%)	0.343
		TC		1 (1.33%)	
	*ITPA* rs1127354	CC	62 (92.5%)	67 (89.3%)	0.509
		CA	5 (7.5%)	8 (10.7%)	
	*ITPA* rs7270101	AA	54 (80.6%)	55 (73.3%)	0.306
		AC	13 (19.4%)	20 (26.7%)	
	*XDH* rs2281547	TT	39 (58.2%)	29 (38.7%)	0.01
		TC	26 (38.8%)	34 (45.3%)	
		CC	2 (3.0%)	12 (16%)	
	ABCB1 rs1045642	AA	7 (10.4%)	2 (2.7%)	0.158
		AG	22 (32.8%)	25 (33.3%)	
		GG	38 (56.7%)	48 (64.0%)	
Allele frequency	TPMT*3C	C	0 (0%)	1 (0.67%)	0.343
	*ITPA* rs1127354	A	5 (3.7%)	8 (5.3%)	0.519
	*ITPA* rs7270101	C	13 (9.7%)	20 (13.3%)	0.34
	*XDH* rs2281547	C	30 (22.3%)	58 (38.6%)	0.003
	*ABCB1* rs1045642	G	98 (73.1%)	121 (80.6%)	0.131

*ABCB1*, ATP Binding Cassette Subfamily B Member 1; *ITPA*, inosine triphosphate pyrophosphatase; TPMT, thiopurine methyltransferase; XDH, Xanthine dehydrogenase.

The reference genotype is indicated first.

**FIGURE 1:**
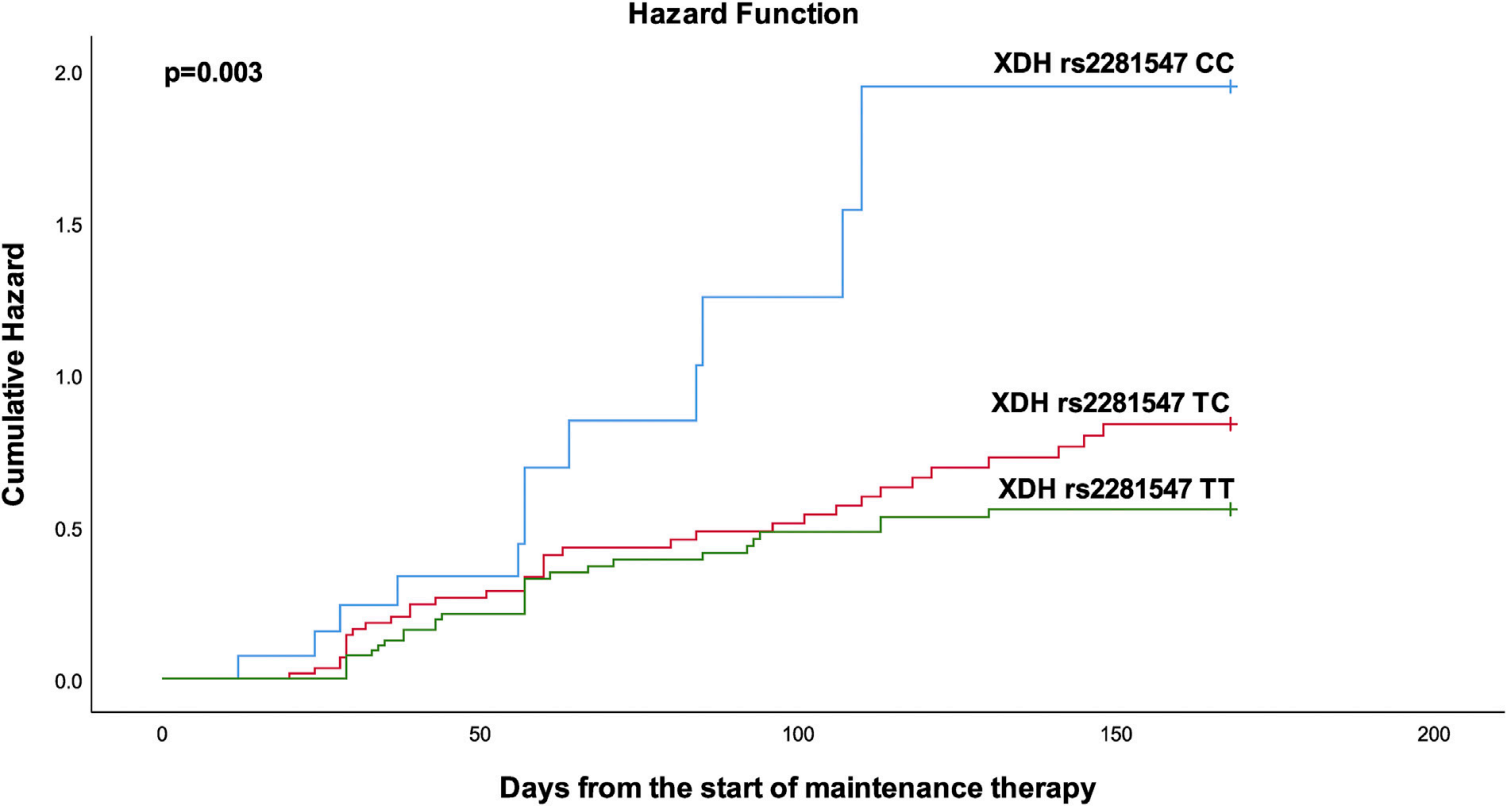
Kaplan-Meier curves to estimate cumulative hazard for the development of Chemotherapy-induced grade 4neutropenia stratified by XDH rs2281547.

Detailed data of cox proportional hazard regression analyses for grade 4 neutropenia are depicted in Table 3. Multivariable analysis showed that patients who carry the *XDH* rs2281547 CC genotype had a higher hazard (AHR 2.956, 95% CI = 1.494–5.849, *p* = 0.002) to develop grade 4 neutropenia than those with the *XDH* rs2281547 TT genotype. The result indicated that *XDH* rs2281547 was an independent genetic predictor of toxicity. Further, Kaplan–Meier hazard curves (Figure 1) show that the risk of developing grade 4 neutropenia increases over time during the first 6 months of maintenance treatment. The cumulative risk of developing grade 4 neutropenia was higher in patients with the CC and TC genotype compared to patients with the TT genotype (*p* = 0.003).

**TABLE 3:** Cox proportional hazard regression results for incidence of grade 4 neutropenia.

SNPs	Bivariable		Multivariable	
	CHR (95% CI)	*p*-value	AHR (95% CI)	*p*-value
*XDH* rs2281547				
TT	1		1	
TC	1.432 (0.872–2.35)	0.156	1.416 (0.86–2.331)	0.171
CC	3.053 (1.550–6.012)	0.001	2.956 (1.494–5.849)	0.002
*ITPA* rs7270101				
AA	1			
AC	1.362 (0.816–2.274)	0.237		
*ITPA* rs1127354				
CC	1			
CA	1.224 (0.588–2.548)	0.589		
*ABCB1* rs1045642				
AA	1			
AG	3.021 (0.715–12.758)	0.133		
GG	3.321 (0.807–13.67)	0.096		

AHR, adjusted hazard ratio; CHR, crude hazard ratio; SNPs, Single nucleotide polymorphisms; XDH, xanthine dehydrogenase; ABCB1, ATP Binding Cassette Subfamily B Member 1; ITPA, inosine triphosphate pyrophosphatase; TPMT, Thiopurine methyltransferase.

All of the variants in bivariable analysis were included in multivariable analysis to see the effects of all the variants for the development of grade 4 neutropenia.

Effects of both genetic and clinical factor on developing grade 4 neutropenia is shown in Supplementary Table S1. Patients with low day 1 maintenance WBC counts were 2.093 times more at risk to develop grade 4 neutropenia than those with normal day 1 maintenance WBC counts. Similarly, patients with age ≤6 years were 2.273 times more at risk to develop grade 4 neutropenia than those who were >6 years of age.

### 3.3 Genotyping and its association with the secondary outcomes

**FIGURE 2:**
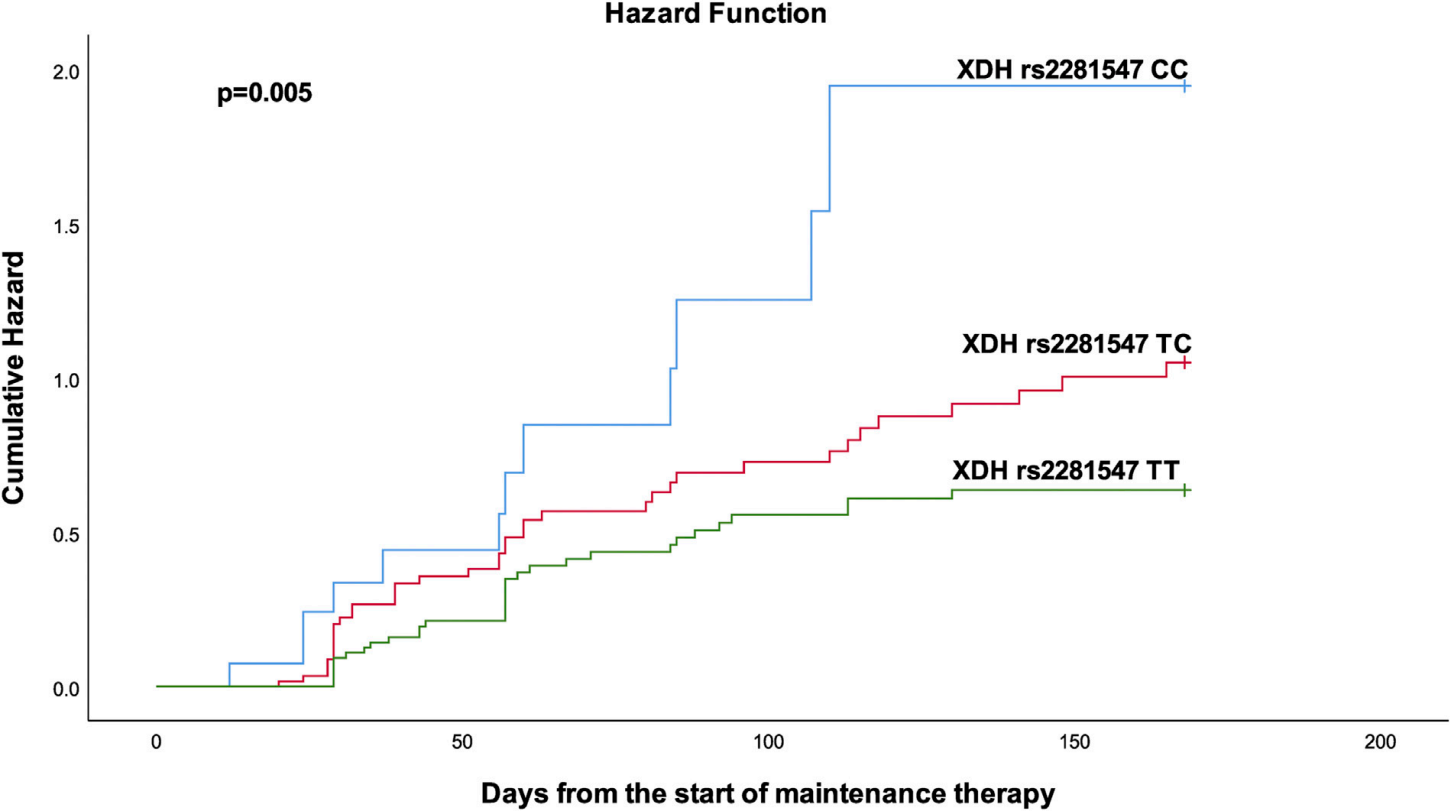
Kaplan-Meier curves to estimate cumulative hazard for treatment interruption stratified by XDH rs2281547.

**FIGURE 3:**
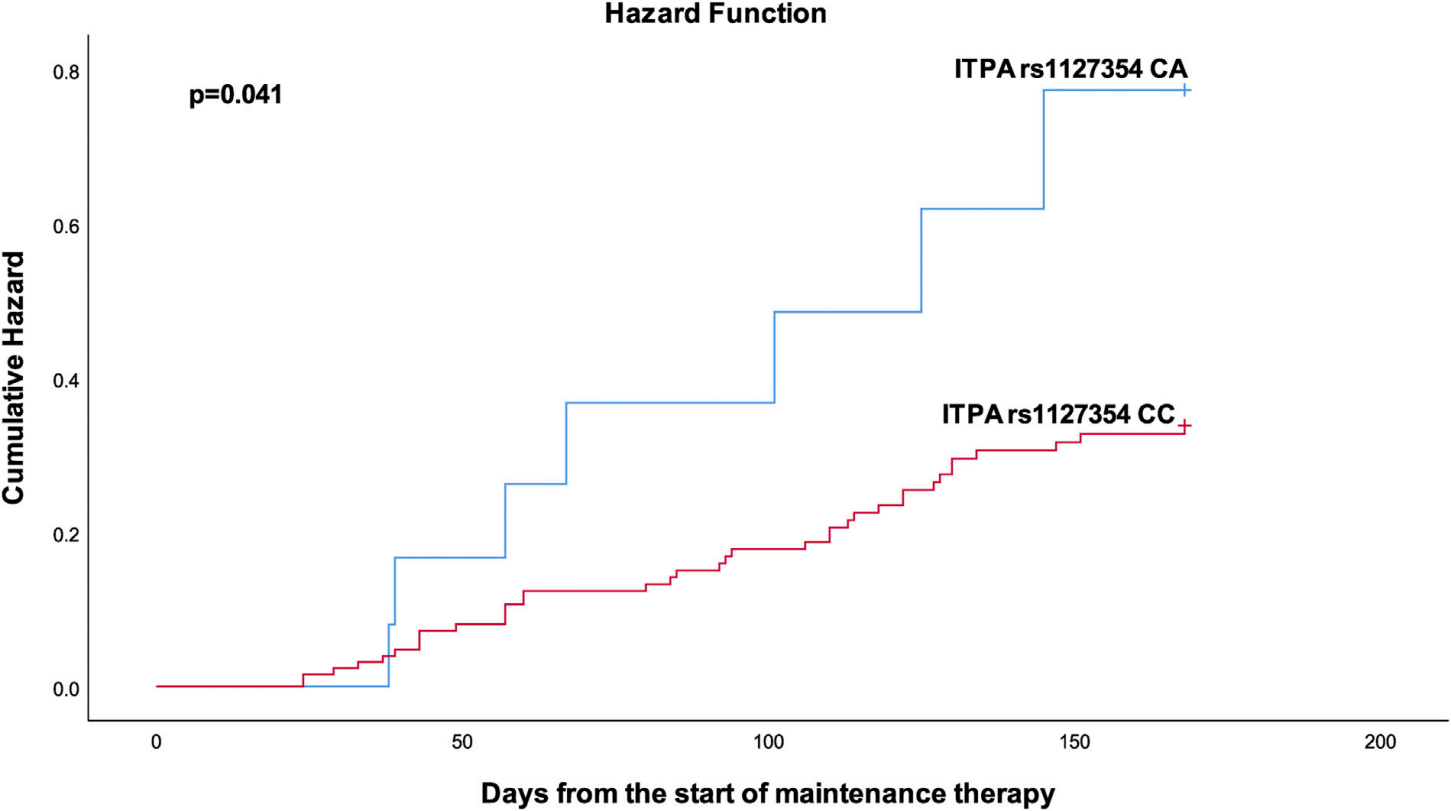
Kaplan-Meier curves to estimate cumulative hazard for neutropenic fever stratified by ITPA rs 1127354.

Table 4 shows the results from the bivariable and multivariable cox regression analyses for the secondary outcomes. Multivariable analysis demonstrated that patients with the *ITPA* rs1127354 AC had a bit more than two-fold (*p* = 0.029) increased risk of developing neutropenic fever. Furthermore, patients with *XDH* rs2281547 TC genotype (AHR = 1.61, 95% CI 1.007–2.573, *p* = 0.047), and CC genotype (AHR 2.704, 95% CI = 1.382–5.289, *p* = 0.004) were also had to experience more treatment interruption than those carrying the TT variant. Kaplan–Meier hazard curves (Figure 2) show that time to treatment interruption hazard was higher in patients with the *XDH* rs2281547 CC and TC genotype compared to persons with the TT genotype (*p* = 0.005). Similarly, the time to neutropenic fever hazard was higher in patients with the *ITPA* rs1127354 AC genotype compared to persons with the CC genotype (*p* = 0.041) (Figure 3).

**TABLE 4:** Cox proportional hazard regression for incidence of neutropenic fever and treatment interruption.

	Neutropenic fever				Treatment interruption			
SNPs	Bivariable		Multivariable		Bivariable		Multivariable	
	CHR (95% CI)	p-value	AHR (95% CI)	p-value	CHR (95% CI)	p-value	AHR (95% CI)	p-value
*XDH* rs2281547								
TT	1				1		1	
TC	1.348 (0.718–2.53)	0.353			1.609 (1.008–2.569)	0.046	1.61 (1.007–2.573)	0.047
CC	1.541 (0.572–4.15)	0.392			2.785 (1.43–5.426)	0.003	2.704 (1.382–5.289)	0.004
*ITPA* rs7270101								
AA	1				1			
AC	1.524 (0.798–2.914)	0.202			1.36 (0.834–2.217)	0.217		
*ITPA* rs1127354								
CC	1		1		1			
CA	2.269 (1.01–5.094)	0.047	2.526 (1.102–5.79)	0.029	1.192 (0.575–2.474)	0.636		
*ABCB1* rs1045642								
AA	1				1			
AG	2.437 (0.317–18.74)	0.392			2.184 (0.662–7.202)	0.199		
GG	3.712 (0.507–27.194)	0.197			2.426 (0.758–7.767)	0.135		

AHR, adjusted hazard ratio; CHR, crude hazard ratio; SNPs, Single nucleotide polymorphisms; XDH, xanthine dehydrogenase, ABCB1 = ATP, Binding Cassette Subfamily B Member 1, ITPA, inosine triphosphate pyrophosphatase; TPMT, Thiopurine methyltransferase.

All of the variants in bivariable analysis were included in multivariable analysis to see the effects of all the variants for the development of grade 4 neutropenia.

Multivariable analysis showed that none of the genetic variants tested were associated with early-onset grade 4 leukopenia (Supplementary Table S2). On the other hand, patients with the *ITPA* rs7270101 AC genotype had about two-fold (*p* = 0.035) increased risk of developing early-onset grade 4 neutropenia.

## 4 Discussion

Inter-individual genetic variations in drug-metabolizing enzymes and transporters affect the toxicity and efficacy of 6-MP, which poses a challenge to the management of patients. Understanding risk factors linked to individual 6-MP intolerance in different ethnicities is vital to facilitate the development of individualized treatment protocol. This is vital for genetic factors as differences in genotype distribution among different ethnic populations limits the predictive value for toxicities. In this study, the incidence of chemotherapy-induced hematologic toxicity and associated risk factors including genetic variations in drug mobilizing enzymes and transporter relevant for the disposition of 6-MP were investigated in childhood ALL patients from Ethiopia.

*NUDT15* rs116855232 and rs746071566, which are considerably lower in frequency in African population, were not detected in this cohort. TPMT*3C is the most prevalent allele among the black population (Ameyaw, 1999; McLeod et al., 1999; Adehin et al., 2017). However, in our cohort, the risk allele frequency of TPMT*3C was 0.35%, about seven times less than described by Ronen *et al.* (Ofri et al., 2010) in Ethiopian Jews. Besides this, other *TPMT* variants were not found at all (*2 and *2A). Thus, the value of predicting hematologic toxicity in this population by genotyping *TPMT* was hindered by the low frequency of well-known variants to be associated with decreased TMPT activity. It is essential to screen a larger group from Ethiopia to determine the exact frequency.

Literature shows that only up to 25% of thiopurine side effects can be explained by TPMT variants (Broekman et al., 2017). Genetic polymorphisms other than *TPMT* such as *ITPA* and *NUDT15* polymorphisms have been shown to be associated with thiopurine toxicity in patients without *TPMT* polymorphisms (Chiengthong et al., 2016; Mao et al., 2021). Recently study linked *XDH* genetic variant with 6-MP induced toxicity. *XDH* contributes to production of the 6-thioxanthine intermediate from 6-MP and it is also involved in the conversion of the intermediate to the final product (Choughule et al., 2014). This is the first study in Ethiopian ALL patients showing that persons homozygous for the minor allele of *XDH* rs2281547 have a higher risk on grade 4 neutropenia. rs2281547 is an intronic variant, which could alter gene expression and yet the functionality of this SNP is not known. Hence, confirmation of the association should be sought in future studies. Poor XDH enzyme activity increases the risk for 6-MP related hematological toxicity, whereas rapid XDH metabolizers have an increased risk of thiopurine failure due to low 6-TGTP formation (Dewit et al., 2010). Co-administration of 6-MP and allopurinol or febuxostat (XDH/XO inhibitors) increased production of active as well as toxic compounds that can lead to severe forms of thiopurine-induced toxicity (Chocair et al., 1993; Dewit et al., 2010). Despite the fact that *XDH* has a role in the metabolism of 6-MP, the potential clinical significance is not yet clear. There are few studies investigating the role of genetic variants in *XDH*. Molybdenum cofactor sulfurase c.362C > T and c.2107C > A variants alter and reduce the metabolic capacity of XDH, slowing thiopurine metabolism that increases formation of the nucleotide 6-thioguanine, which is hematotoxic (Kurzawski et al., 2012; Stiburkova et al., 2018). A recent pathway genes association study reported the role of *XDH* in thiopurine-related toxicities; neutropenia, hepatotoxicity, and treatment interruption (Choi et al., 2019). A case report study also identified that hematotoxicity caused by azathioprine is aggravated by xanthine oxidase deficiency (Serre-Debeauvais et al., 1995).

This study also identified associations between ITPA polymorphisms and 6-MP related hematological toxicity. The minor allele frequency of *ITPA* rs1127354 and *ITPA* rs7270101 was 4.6%, and 11.6%, respectively, which is similar to other studies (William et al., 2011; Smid et al., 2014; Hareedy et al., 2015). *ITPA* acts as a house-cleaning enzyme since it hydrolyzes 6-TITP back to 6-TIMP, thus preventing the accumulation of 6-TITP, which is toxic (Zamzami et al., 2013; Barba et al., 2022). A recent meta-analysis suggested that populations with low TPMT and high ITPA variant allele frequencies, such as Asians are more susceptible to the influence of ITPA variants (Barba et al., 2022). In the present study, the *TPMT* allele frequencies were also low and interestingly *ITPA* rs1127354 was significantly associated with the incidence of febrile neutropenia. This is in agreement with a previous study (Stocco et al., 2009), showing a significantly higher probability of severe febrile neutropenia in patients with a variant in *ITPA* among patients who received a *TPMT* genotype-guided dose of 6-MP. As reported in an Egyptian ALL study, *ITPA* rs7270101 was associated with the risk of neutropenia and leukopenia (Hareedy et al., 2015). In agreement with these results, we show that *ITPA* rs7270101 was associated with neutropenia that developed within the initial 60 days of the maintenance therapy. This is suggesting that *ITPA* rs7270101 could be an important genetic predictor for determining the 6-MP dose intensity with the risk allele carriers requiring a low 6-MP dose. This is the first study in Ethiopian population showing that *ITPA* alleles are associated with 6-MP related hematological toxicity during maintenance therapy. Nevertheless, there is no consensus on the impact of genetic polymorphisms in ITPA on thiopurine induced hematologic toxicity yet and its application in a clinic setting has not been unified, especially in different ethnic populations (Zhou et al., 2018; Mao et al., 2021).

This study showed that a higher incidence of grade 4 hematologic toxicities (mainly manifested as neutropenic toxicity) caused treatment interruption (Table 1). The finding of this study revealed that *XDH* rs2281547 are strong genetic predictor of grade 4 hematologic toxicities in this cohort of Ethiopian children. This study provides first information on genetic factors associated with hematologic toxicities in Ethiopian childhood ALL patients. However, the outcome of the current study is limited as only five genes in thiopurine pathway were tested. Other limitations include small sample size, a single institutional study, and lack of validation cohorts.

## 5 Conclusion

In the current study, the rate of hematological toxicity was high. XDH and ITPA variants were associated with grade 4 hematologic toxicities during maintenance therapy. TPMT*3C variant frequency was very low in this cohort. This study adds knowledge on how to identify pediatric ALL patients at high risk for chemotherapy induced toxicity in a population from Ethiopia. In the future, it is essential to study large groups of patients from different ethnic background to determine which genes are important to predict grade 4 hematological toxicities in different ethnic populations.

## Data availability statement

The original contributions presented in the study are included in the article/Supplementary Materials, further inquiries can be directed to the corresponding author.
