# Supplementary material for: Genetic variants of genes involved in thiopurine metabolism pathway are associated with 6-mercaptopurine toxicity in pediatric acute lymphoblastic leukemia patients from Ethiopia
Source: Front Pharmacol. 2023 May 9;14:1159307. doi: 10.3389/fphar.2023.1159307 (PMC10214954; doi:10.3389/fphar.2023.1159307)
Supplement: Supplementary file 1 [file Table1.docx]

Supplementary

Supplementary Table 1. Cox proportional hazard regression results for incidence of grade 4 neutropenia

| Predictor Factor | Bivariable | | Multivariable | |
| --- | --- | --- | --- | --- |
|  | CHR (95% CI) | *p*-value | AHR (95% CI) | *p*-value |
| Child’s age (Years)  > 6  ≤ 6 | 1  1.891 (1.174-3.045) | 0.009 | 1  2.273 (1.395-3.704) | 0.001 |
| Maintenance day 1 WBC  ≥4500  <4500 | 1  2.155 (1.28-3.63) | 0.004 | 1  2.093 (1.223-3.580) | 0.007 |
| *XDH* rs2281547  TT  TC  CC | 1  1.432 (0.872-2.35)  3.053 (1.550-6.012) | 0.156  0.001 | 1  1.493 (0.906-2.460)  2.481 (1.247-4.934) | 0.116  0.01 |
| *ABCB1* rs1045642  AA  AG GG | 1  3.021 (0.715-12.758)  3.321 (0.807-13.67) | 0.133  0.096 |  |  |

_ANC = Absolute neutrophil count, AHR = Adjusted hazard ratio, CHR = Crude hazard ratio WBC = White blood cell count,_ *_XDH_* _= Xanthine dehydrogenase. It was tested whether the independent variables (child’s age, sex, risk group, WBC and genotype) could predict the outcome (grade 4 neutropenia). Factors with a p<0.2 in bivariable are depicted in the table and included in the multivariable analysis to see the influence of both clinical and genetic factor._

Supplementary Table 2. Cox proportional hazard regression for predictors of the early-onset grade 4 leukopenia and neutropenia

| **SNPs** | **Early-onset grade 4 leukopenia** | | | | **Early-onset grade 4 neutropenia** | | | | |
| --- | --- | --- | --- | --- | --- | --- | --- | --- | --- |
|  | **Bivariable** | | **Multivariable** | | **Bivariable** | | **Multivariable** | | |
|  | **COR (95% CI)** | ***p*-value** | **AOR (95% CI)** | ***p*-value** | **COR (95% CI)** | ***p*-value** | **AOR (95% CI)** | ***p*-value** | |
| ***XDH* rs2281547**  **TT**  **TC**  **CC** | **1**  **1.838 (0.795-4.246)**  **3.019 (1.012-9.012)** | **0.154**  **0.048** |  |  | **1**  **1.267 (0.676-2.375)**  **2.170 (0.912-5.164)** | **0.46**  **0.08** |  | |  |
| ***ITPA* rs7270101**  **AA**  **AC** | **1**  **0.856 (0.347-2.111)** | **0.736** |  |  | **1**  **1.825 (0.995-3.349)** | **0.052** | **1**  **1.926 (1.046-3.547)** | | **0.035** |
| ***ITPA* rs1127354**  **CC**  **CA** | **1**  **1.184 (0.358-3.923)** | **0.782** |  |  | **1**  **0.921 (0.33-2.567)** | **0.874** |  | |  |
| ***ABCB1* rs1045642**  **AA**  **AG GG** | **1**  **1.447 (0.178-11.762)**  **2.343 (0.314-17.465)** | **0.73**  **0.406** |  |  | **1**  **3.088 (0.406-23.48)**  **3.972 (0.542-29.10)** | **0.276**  **0.175** |  | |  |

_AHR = Adjusted odds ratio, CHR = Crude odds ratio, SNPs =Single nucleotide polymorphisms,_ *_XDH_* _= Xanthine dehydrogenase,_ *_ABCB1 =_* _ATP Binding Cassette Subfamily B Member 1,_ *_ITPA_* _= Inosine triphosphate pyrophosphatase,_ *_TPMT_* _= Thiopurine methyltransferase. All of the variants in bivariable analysis were included in multivariable analysis to see the effects of all the variants for the development of early-onset grade 4 leukopenia/neutropenia._
